# Optimal Time Allocation in Backscatter Assisted Wireless Powered Communication Networks

**DOI:** 10.3390/s17061258

**Published:** 2017-06-01

**Authors:** Bin Lyu, Zhen Yang, Guan Gui, Hikmet Sari

**Affiliations:** Key Laboratory of Ministry of Education in Broadband Wireless Communication and Sensor Network Technology, Nanjing University of Posts and Telecommunications, Nanjing 210003, China; 13010511@njupt.edu.cn (B.L.); guiguan@njupt.edu.cn (G.G.); hsari@ieee.org (H.S.)

**Keywords:** wireless powered communication network, backscatter communication, harvest-then-transmit mode, optimal time allocation policy

## Abstract

This paper proposes a wireless powered communication network (WPCN) assisted by backscatter communication (BackCom). This model consists of a power station, an information receiver and multiple users that can work in either BackCom mode or harvest-then-transmit (HTT) mode. The time block is mainly divided into two parts corresponding to the data backscattering and transmission periods, respectively. The users first backscatter data to the information receiver in time division multiple access (TDMA) during the data backscattering period. When one user works in the BackCom mode, the other users harvest energy from the power station. During the data transmission period, two schemes, i.e., non-orthogonal multiple access (NOMA) and TDMA, are considered. To maximize the system throughput, the optimal time allocation policies are obtained. Simulation results demonstrate the superiority of the proposed model.

## 1. Introduction

With the development of Internet of Things (IoT), wireless sensor networks (WSNs) have received a lot of attention [1,2,3,4,5,6,7,8,9,10]. There are many research directions focusing on WSNs, such as security [2,3,4,5,6] and wireless power transfer (WPT) [7,8,9,10]. The security is an important issue of WSNs since wireless sensors typically suffer from different types of attacks. To improve the security of WSNs, some novel schemes have been proposed [2,3,4,5,6]. For example, an active detection-based security and trust routing scheme was proposed in [3] to avoid black hole attack and improve the data route success probability. Moreover, due to the inherent resource-constrained characteristics of wireless sensors, how to prolong the lifetime of these sensors is also an important issue. Concurrently, WPT has been identified as a potential solution to this issue [7].

The existing WPT techniques are typically categorized into three types, i.e., inductive coupling, magnetic resonant coupling and electromagnetic (EM) radiation [8]. Due to the demand for far-field WPT, radio frequency (RF)-based WPT techniques exploiting radiative properties of EM waves have been a hot topic. With the development of RF-based WPT techniques, wireless powered communication networks (WPCNs) have been proposed [9,10]. In a WPCN, there are typically a hybrid access point (HAP) with stable energy sources and multiple users with supercapacitors. To make this network operate, the “harvest-then-transmit” (HTT) mode including energy harvesting and data transmission periods was proposed in [9]. The users first harvest energy radiated by the HAP in the downlink (DL) during the energy harvesting period and then transmit data to the HAP in the uplink (UL) using the harvested energy during the data transmission period. In [9], the WPT in the DL and wireless information transmission (WIT) in the UL work in the half duplex (HD) mode since the HAP has only one antenna. To improve the system performance, a full duplex (FD)-WPCN was proposed by employing two antennas at the HAP [10]. In [10], the HAP broadcasts energy to all users over the entire block so that the users could use the energy harvested before their data transmission. In both [9] and [10], only the HTT mode was adopted so that only a part of a time block was used for information transmission, which limited the system performance.

To extend the time for information transmission, backscatter communication (BackCom) [11,12] has received a lot of attention [13,14,15]. Typically, BackCom can be categorized into three types, i.e., monostatic backscatter, bistatic scatter and ambient backscatter [16,17,18]. In BackCom systems, the users backscatter modulated signals to the information receiver based on the instantaneous excitation signals such that the dedicated time for energy harvesting is not required. In [13], a backscatter assisted WPCN (BAWPCN) was proposed. In a BAWPCN, there are two types of users, which can work in BackCom and HTT modes, respectively. The users working in the BackCom mode are scheduled during the energy harvesting period of the HTT mode. To make the network deployment flexible, the authors in [14] assumed that a user could work in either BackCom mode or HTT mode. A single-user cognitive WPCN (a special type of WPCNs) was considered and a tradeoff between the BackCom and HTT modes was analyzed. Based on [14], the authors in [15] further considered a multi-user case and the optimal time allocation policy was studied. In [14,15], the overlay-based cognitive WPCNs were studied so that the data transmission period of the HTT mode was fixed. Moreover, the energy source was only available when the primary channel was busy, which limited the system flexibility.

**Figure 1:**
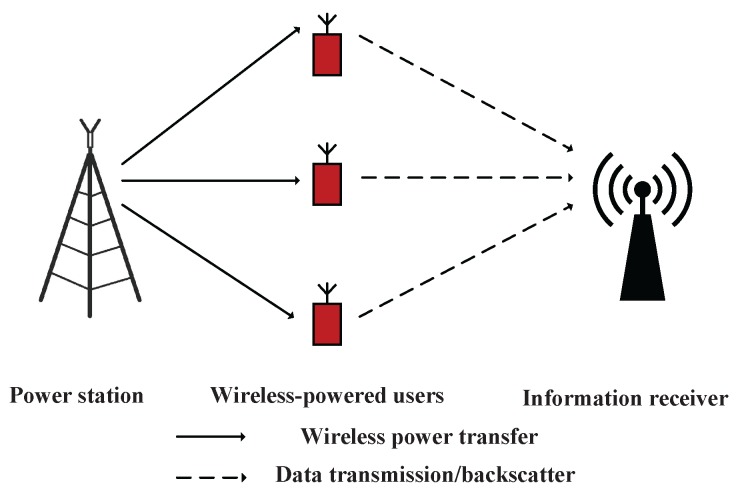
Model of a backscatter assisted wireless powered communication network (BAWPCN).

Different with [14,15], in this paper, we consider a general BAWPCN, which consists of a power station, an information receiver, and multiple users that can work in either BackCom mode or HTT mode, which is shown in Figure 1. The proposed model is studied based on a time block. The time block is mainly divided into two parts corresponding to the data backscattering period of the BackCom mode and the data transmission period of the HTT mode. The power station broadcasts wireless energy to all users continuously over the entire block. During the data backscattering period, the users backscatter data to the information receiver in time division multiple access (TDMA). Note that the data backscattering period is partly overlaid with the energy harvesting period. When one user works in the BackCom mode, the other users stay in the HTT mode to harvest energy. During the data transmission period, we consider two schemes, i.e., non-orthogonal multiple access (NOMA) [19,20,21] and TDMA. We consider the NOMA scheme for the improvement of spectral efficiency and user fairness [19,21], and the TDMA scheme for the improvement of sum-throughput since the full-duplex mode is adopted [10]. The key contributions of this paper are summarized as follows. First, we propose a general WPCN assisted by BackCom, where both BackCom and HTT modes are employed at the users. The time is fully exploited for information transmission. Second, both NOMA and TDMA schemes are considered during the data transmission period of the HTT mode. It is shown that the TDMA scheme can provide higher system throughput than the NOMA scheme. Third, the optimal time allocation policies are derived to maximize the system throughput.

## 2. System Model

As illustrated in Figure 1, we consider a BAWPCN that consists of one power station, one information receiver, and *K* wireless-powered users without fixed energy supplies. Each user, denoted by $U_{i},i=1,\cdots ,K$ , has an energy harvesting module and a backscatter circuit so that it can work in either HTT mode or BackCom mode [14,15]. It is highlighted that the BackCom and HTT modes cannot be operated simultaneously [22]. The power station continuously broadcasts energy signals to all users. When the HTT mode is adopted, the users harvest energy from the power station and store the harvested energy in their supercapacitors for further data transmission. In contrast, when the BackCom mode is activated, the users can backscatter the modulated signals to the information receiver instantaneously. It is assumed that the information receiver has the perfect information of each user’s working mode and can choose the corresponding demodulators to extract useful information [14,15].

The channel power gains from the power station to $U_{i}$ , and from $U_{i}$ to the information receiver are denoted as $h_{i}$ and $g_{i}$ , respectively. We assume the channels are quasi-static flat fading and remain constant during each block with duration *T*. Moreover, it is assumed that all terminals perfectly know the channel power gains.

**Figure 2:**
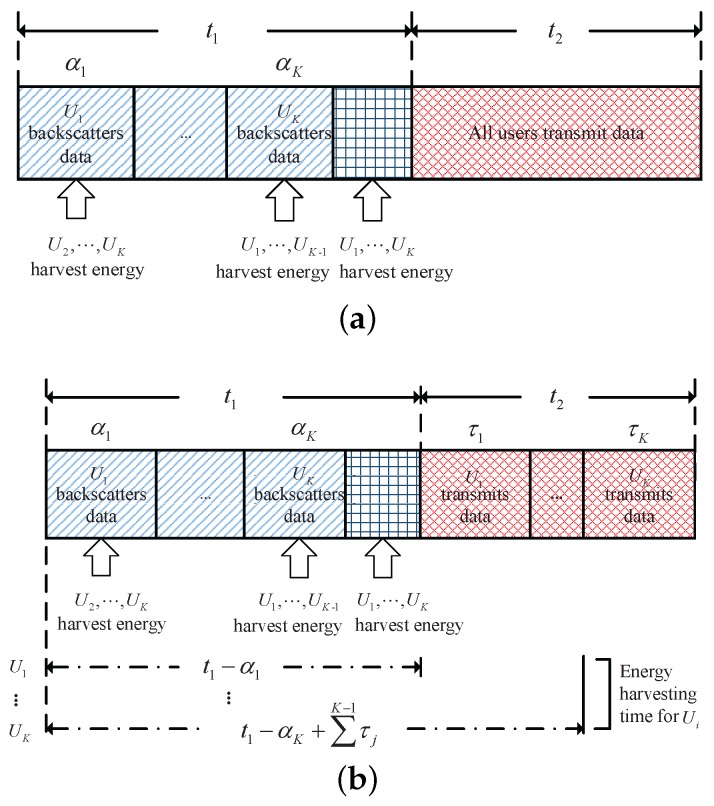
Block structure of the BAWPCNs. (**a**) non-orthogonal multiple access (NOMA) scheme; (**b**) time division multiple access (TDMA) scheme.

The block structure for the proposed WPCN system is shown in Figure 2. Each block is divided into two main parts corresponding to the data backscattering and transmission periods, respectively. The duration of the two periods are denoted by $t_{1}$ and $t_{2}$ . During the data backscattering period, $U_{i}$ backscatters data to the information receiver during its allocated time slot $\alpha_{i}$ , whereas, the other users harvest energy from the power station [15]. The total time for energy harvesting of $U_{i}$ during the data backscattering period is given as $t_{1}-\alpha_{i}$ . During the data transmission period, we consider two schemes for data transmission, i.e., NOMA and TDMA.

### 2.1. BackCom Mode

In this paper, we consider one type of BackCom, i.e., ambient backscatter [17]. For BackCom, $U_{i}$ can harvest energy from the signals transmitted by the power station even if it is backscattering data to the information receiver. However, the harvested energy is not sufficient for $U_{i}$ to transmit data actively but can supply the operations of $U_{i}$ . Hence, we do not discuss the BackCom mode’s circuit energy consumption here. From [17], the rate of ambient backscatter is controlled by the setting of RC circuit elements. Denote the backscattering rate of $U_{i}$ as $B_{i}$ , which is a constant. Hence, the total transmission bits of $U_{i}$ during the backscattering data period is

(1) $$\begin{array}{c} R_{i}^{b}=\alpha_{i}B_{i}. \end{array}$$

### 2.2. HTT Mode

Since different schemes are considered during the data transmission period, the energy harvesting and data transmission periods of each scheme are different. The details are given as follows.

#### 2.2.1. NOMA Scheme

For the NOMA scheme, all users transmit data to the information receiver simultaneously and SIC is employed at the information receiver to retrieve the users’ signals. To guarantee the performance of NOMA scheme, it is assumed that the channel power gains of users are distinct. The indices of these users are assigned in a way that $h_{i}g_{i}$ are sorted in descending order, i.e., $h_{1}g_{1}>h_{2}g_{2}>\cdots >h_{K}g_{K}$ [21,23]. The energy harvesting and data transmission periods of $U_{i}$ are given as $t_{1}-\alpha_{i}$ and $t_{2}$ , respectively. The total harvested energy of $U_{i}$ , denoted by $E_{i}$ , is given by

$$E_{i}^{h}=\eta_{i}\left(t_{1}-\alpha_{i}\right)Ph_{i},$$

where $\eta_{i}$ is the energy harvesting efficiency, *P* is the transmit power of the power station. Following the assumptions in [9], we assume that the main energy consumption during the data transmission period is due to the data transmission, and thus the circuit energy consumption is ignored here for simplicity. Moreover, we assume that the harvested energy will be exhausted during the data transmission period. Denote the average transmit power of $U_{i}$ during the data transmission period as $P_{i}^{t}$ , which is given by

$$P_{i}^{t}=\frac{E_{i}^{h}}{t_{2}}.$$

The transmission bits of $U_{i}$ , denoted by $R_{i}^{n}$ , are expressed as

(2) $$\begin{array}{ccc} R_{i}^{n} & = & t_{2}W\log_{2}\left(1+\frac{P_{i}^{t}g_{i}}{\sum_{j=i+1}^{K}P_{j}^{t}g_{j}+\sigma^{2}}\right)\\ & = & t_{2}W\log_{2}\left(1+\frac{\frac{t_{1}-\alpha_{i}}{t_{2}}\gamma_{i}}{\sum_{j=i+1}^{K}\frac{t_{1}-\alpha_{j}}{t_{2}}\gamma_{j}+1}\right),\;i=1,\cdots ,K-1, \end{array}$$

(3) $$R_{K}^{n}=t_{2}W\log_{2}\left(1+\frac{t_{1}-\alpha_{K}}{t_{2}}\gamma_{K}\right).$$

where $\gamma_{i}=\frac{\eta_{i}Ph_{i}g_{i}}{\sigma^{2}}$ .

#### 2.2.2. TDMA Scheme

For the TDMA scheme, the users transmit data to the information receiver sequentially. Since the power station broadcasts energy during the entire block, when $U_{i}$ is transmitting data to the information receiver, other users can still harvest energy from the power station. Due to the high self-discharge of the supercapacitors, we assume that $U_{i}$ can harvest energy before its data transmission but not after [10]. Hence, compared with the NOMA scheme, the users in this scheme can harvest more energy, which provides much larger system throughput. We denote the time for $U_{i}$ to transmit data as $\tau_{i}$ . The number of total energy harvested by $U_{i}$ is given by

$$E_{i}=\eta_{i}\left(t_{1}-\alpha_{i}+\sum_{j=1}^{i-1}\tau_{j}\right)Ph_{i},\;i=1,\cdots ,K,$$

where $t_{1}-\alpha_{i}+\sum_{j=1}^{i-1}\tau_{j}$ is the energy harvesting time for $U_{i}$ , and let $\sum_{j=1}^{0}\tau_{j}=0$ .

Since the users transmit data to the information receiver in TDMA, there is no mutual interference among these users. Hence, the transmission bits of $U_{i}$ are given by

(4)Rit=τiWlog2(1+Eigiτiσ2)(5)=τiWlog2(1+t1-αi+∑j=1i-1τjτiγi),i=1,⋯,K.

To simplify description, we refer to the NOMA scheme of the HHT mode and to the TDMA scheme of the HTT mode in a BAWPCN as NOMA-BAWPCN and TDMA-BAWPCN, respectively.

## 3. Sum-Throughput Maximization

In this section, we study the sum-throughput maximization problems of both NOMA-BAWPCN and TDMA-BAWPCN. The optimization problems are first formulated and the optimal time allocation policies are then studied. For the NOMA-BAWPCN, the formulation of the total transmission bits of both BackCom and HTT modes is given by

(6) $$\begin{array}{ccc} R_{\mathrm{sum}} & = & \sum_{i=1}^{K}R_{i}^{b}+\sum_{i=1}^{K}R_{i}^{n}\\ & = & \sum_{i=1}^{K}\alpha_{i}B_{i}+t_{2}W\log_{2}\left(1+\sum_{i=1}^{K}\frac{\left(t_{1}-\alpha_{i}\right)\gamma_{i}}{t_{2}}\right)-t_{2}W\log_{2}\left(1+\sum_{i=2}^{K}\frac{\left(t_{1}-\alpha_{i}\right)\gamma_{i}}{t_{2}}\right)\\ & + & t_{2}W\log_{2}\left(1+\sum_{i=2}^{K}\frac{\left(t_{1}-\alpha_{i}\right)\gamma_{i}}{t_{2}}\right)-t_{2}W\log_{2}\left(1+\sum_{i=3}^{K}\frac{\left(t_{1}-\alpha_{i}\right)\gamma_{i}}{t_{2}}\right)+\cdots \\ & + & t_{2}W\log_{2}\left(1+\sum_{i=K-1}^{K}\frac{\left(t_{1}-\alpha_{i}\right)\gamma_{i}}{t_{2}}\right)-t_{2}W\log_{2}\left(1+\frac{\left(t_{1}-\alpha_{K}\right)\gamma_{K}}{t_{2}}\right)+t_{2}W\log_{2}\left(1+\frac{\left(t_{1}-\alpha_{K}\right)\gamma_{K}}{t_{2}}\right)\\ & = & \sum_{i=1}^{K}\alpha_{i}B_{i}+t_{2}W\log_{2}\left(1+\frac{\sum_{i=1}^{K}\left(t_{1}-\alpha_{i}\right)\gamma_{i}}{t_{2}}\right). \end{array}$$

Denote $\alpha =[\alpha_{1},\cdots ,\alpha_{K}]$ and $t=[t_{1},t_{2}]$ . From the time allocation policy given in the previous sections, we derive the following constraints

$$\begin{array}{c} C1\text{:}\;\sum_{i=1}^{K}\alpha_{i}\leq t_{1},\\ C2\text{:}\;t_{1}+t_{2}\leq T,\\ C3\text{:}\;\alpha_{i}\geq 0,i=1,\cdots ,K,\\ C4\text{:}\;t_{1},t_{2}\geq 0. \end{array}$$

C1 limits the total time of all users in the BackCom mode, C2 ensures that two main parts cannot exceed the duration of a block, C3 and C4 are the non-negative orthant constraints for the time allocation variables.

Based on the above analysis, the optimization problem of the NOMA-BAWPCN is formulated as

$$\begin{array}{cc} \left(P1\right)\;\max_{\alpha ,t}\;\;\; & R_{\mathrm{sum}}\\ s.t.\; & C1,C2,C3,C4. \end{array}$$

**Lemma** **1.**
$R_{sum}\left(\alpha ,t\right)$ *is a concave function of* α *and* t .

**Proof.**  Please refer to Appendix A.  ☐

Moreover, all constraints of Problem P1 are affine. Hence, we derive that Problem P1 is a convex optimization problem [24]. There are many standard numerical methods which can be used to solve a convex problem. To make the implementation simple, we adopt the interior-point method [24].

For the TDMA-BAWPCN, the expression of the total transmission bits is given by

(7) $$\begin{array}{ccc} R_{\mathrm{sum}} & = & \sum_{i=1}^{K}R_{i}^{b}+\sum_{i=1}^{K}R_{i}^{t}\\ & = & \sum_{i=1}^{K}\left[\alpha_{i}B_{i}+\tau_{i}W\log_{2}\left(1+\frac{t_{1}\;-\;\alpha_{i}\;+\;\sum_{j=1}^{i-1}\tau_{j}}{\tau_{i}}\gamma_{i}\right)\right]. \end{array}$$

In addition to the constraints given for the NOMA-BAWPCN, the TDMA-BAWPCN also follows the following constraints

$$\begin{array}{c} C5\text{:}\;\sum_{i=1}^{K}\tau_{i}\leq t_{2},\\ C6\text{:}\;\tau_{i}\geq 0,\;i=1,\cdots ,K. \end{array}$$

C5 limits the total time for data transmission in the HTT mode, and C6 also guarantees that the variables are non-negative. Denote $\tau =[\tau_{1},\cdots ,\tau_{K}]$ . The optimization problem for the TDMA-BAWPCN is formulated as

$$\begin{array}{cc} \left(P2\right)\;\max_{\alpha ,\tau ,t}\;\;\; & R_{\mathrm{sum}}\\ s.t.\; & C1,C2,C3,C4,C5,C6. \end{array}$$

Taking the method used for Problem P1, we can also derive that Problem P2 is a convex optimization problem. Hence, the interior-point method is adopted.

## 4. Simulation Results

In this section, simulation results are presented to evaluate the performance of the proposed model. We take a similar setting of parameters as given in [9]. We set the transmission bandwidth as 100 kHz, the energy harvesting efficiency as $\eta_{i}=0.6,\forall i$ , the noise power at the information receiver as $\sigma^{2}=-90$ dBm, and T=1 s. The forward and backward channel power gains are modelled as $h_{i}=10^{-3}d_{fi}^{-3}\rho_{fi}^{2}$ and $g_{i}=10^{-3}d_{bi}^{-3}\rho_{bi}^{2}$ , where $d_{fi}$ is the distance between $U_{i}$ and the power station, $d_{bi}$ is the distance between $U_{i}$ and the information receiver, $\rho_{fi}$ and $\rho_{bi}$ are the channel short-term fadings, $\rho_{fi}^{2}$ and $\rho_{bi}^{2}$ are exponentially distributed random variables with unit mean. Let $d_{fi}=4+\frac{i}{K}*4$ m and $d_{bi}=3+\frac{i}{K}*3$ m, which can guarantee that the channel power gains of users are distinct [23]. Unless otherwise stated, the backscatter rate and transmit power are set as 300 kbps and 30 dBm, respectively. For performance comparison, the scheme that all users only work in the BackCom mode and the scheme that all users only work in the HTT mode are used as benchmarks. All simulation curves are obtained by averaging 1000 Monte Carlo runs.

**Figure 3:**
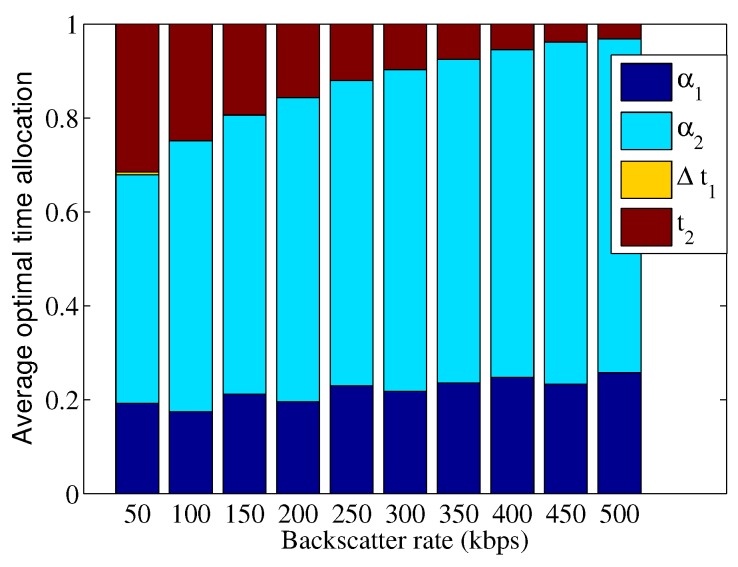
Average optimal time allocation versus different backscatter rates for NOMA-BAWPCNs.

**Figure 4:**
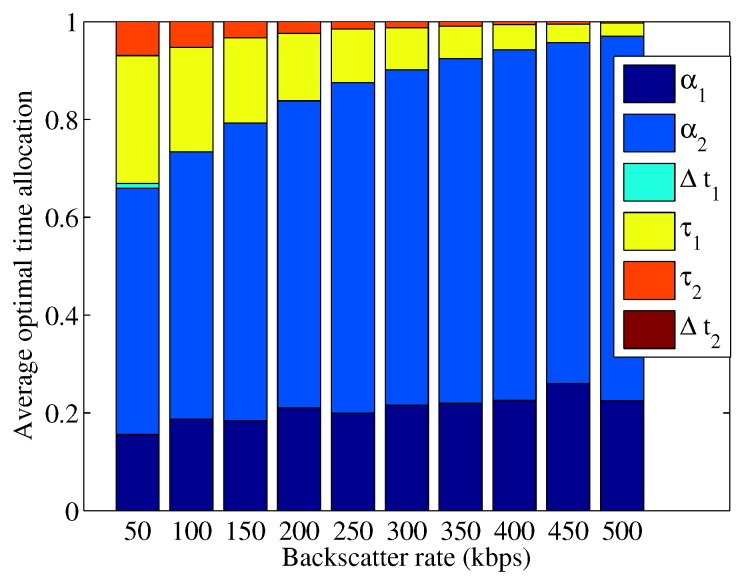
Average optimal time allocation versus different backscatter rates for TDMA-BAWPCNs.

We first consider a two-user BAWPCN, i.e., K=2 . To show the effect of backscatter rate on time allocation, the following case, i.e. the backscatter rates of both two users are the same and varied from 50 kbps to 500 kbps, is studied. Let $\Delta t_{1}=t_{1}-\alpha_{1}-\alpha_{2}$ and $\Delta t_{2}=t_{2}-\tau_{1}-\tau_{2}$ , which correspond to the dedicated time for energy harvesting for both two users and the unused time for data transmission, respectively. The results of average optimal time allocation are given in Figure 3 and Figure 4. From Figure 3 and Figure 4, we observe that the total time used for data backscattering increases with the increase of backscatter rate. This result can be explained as follows. For the BackCom mode with an increasing rate, increasing the backscattering time can provide higher transmission bits and increase the energy harvested by users. In addition, since the total time for users to transmit data is reduced, users can transmit data with a higher transmit power. Overall, the total transmission bits improve. Compared with $U_{2}$ , $U_{1}$ has more time to harvest energy since it has a better channel condition. Moreover, $\Delta t_{2}$ is always equal to zero since $t_{2}$ will be fully exploited to transmit data.

**Figure 5:**
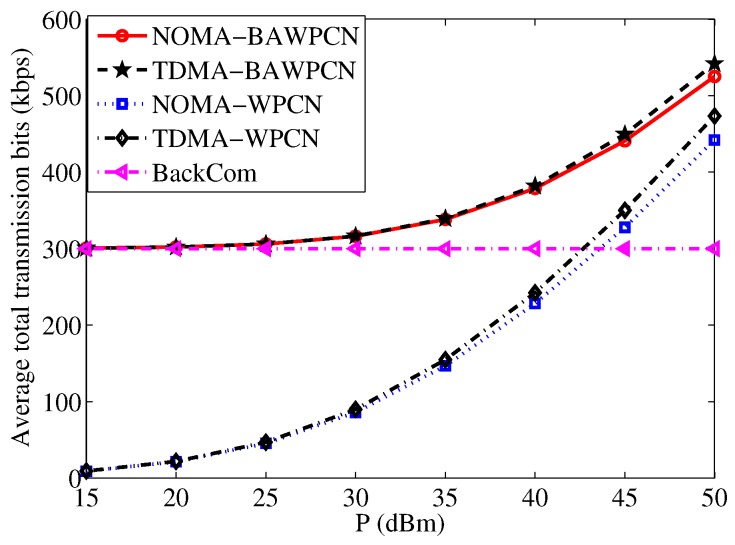
Average total transmission bits versus transmit power.

For the two-user case, we further show the effect of transmit power on total transmission bits in Figure 5. As the transmit power increases, the total-transmission bits of both WPCN and BAWPCN models improve. When the transmit power is small, the performance of the BAWPCNs is similar to that of the BackCom mode, but far better than that of the WPCNs. When the transmit power is large, the performance gap between the BAWPCNs and WPCNs reduces. This is because the HTT mode begins to dominate the total transmission bits with a higher transmit power. It is emphasized that the proposed model with corresponding data transmission schemes always achieves a better performance than the traditional models, e.g., the total transmission bits of the NOMA-BAWPCN are larger than that of the NOMA-WPCN. In addition, for the proposed model, the TDMA scheme can achieve larger total transmission bits than the NOMA scheme since the TDMA scheme can harvest more energy resulting in higher transmission bits for the HTT mode.

**Figure 6:**
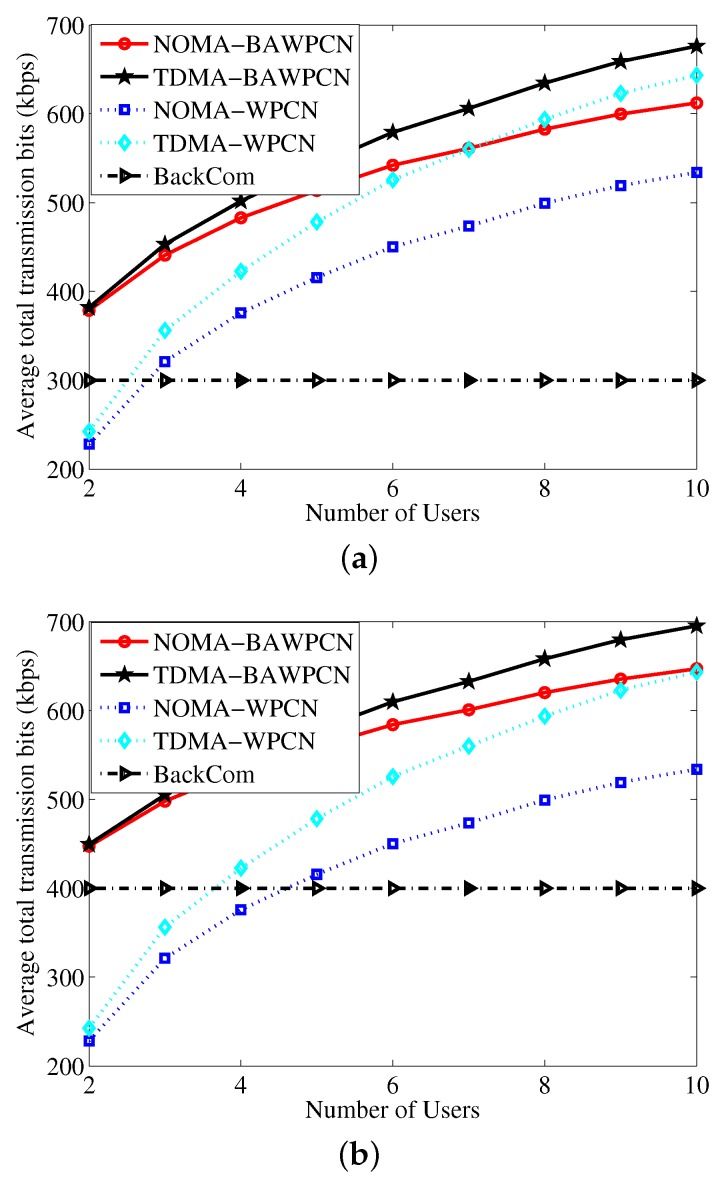

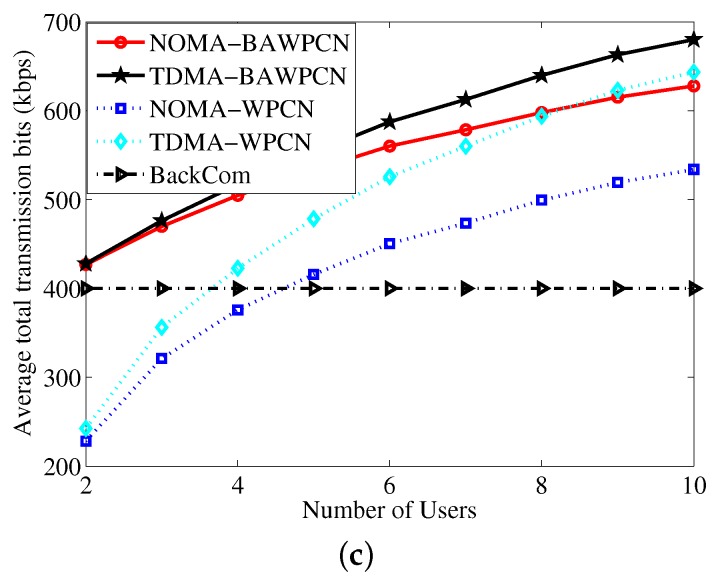
Average total transmission bits versus number of users. (**a**) Case (i); (**b**) Case (ii); (**c**) Case (iii).

We investigate the average total transmission bits versus the number of users in Figure 6 by considering the following three cases. Case (i): All users have the same backscatter rate which is fixed as 300 kbps; Case (ii): One user’s backscatter rate is 300 kbps and the other users’ backscatter rate is 400 kbps. Case (iii): One user’s backscatter rate is 400 kbps and the other users’ backscatter rate is 300 kbps. From Figure 6, we observe the average total transmission bits of the BAWPCNs and WPCNs are increasing functions of the number of users. As the number of users increases, the total transmission bits of the TDMA-WPCN may be larger than that of the NOMA-BAWPCN. This is because the users of the TDMA-WPCN can harvest more energy, especially when the number of users is large enough. Compared with Figure 6a, the performance of BAWPCNs in Figure 6b,c is better. This is because at least one user in Case (ii) and Case (iii) has a larger backscatter rate. Moreover, the performance of BAWPCNs in Case (ii) is superior to that in Case (iii). This result implies that the performance depends on not only the largest backscatter rate but also on the number of users with the largest backscatter rate, which is different from the result in [15]. From Figure 6, we also observe that the proposed model with corresponding data transmission schemes can achieve larger total transmission bits and the total transmission bits of the TDMA-BAWPCN are the largest, which is coincident with the observation in Figure 5.

## 5. Conclusions

This paper has studied a backscatter assisted wireless powered communication network, where both BackCom and HTT modes are employed at the users. During the data transmission period, both NOMA and TDMA schemes have been considered. To maximize the system throughput, optimization problems have been formulated and the time allocation policies including the tradoffs between data backscattering and energy harvesting, and between data backscattering and data transmission have been studied. Simulation results have confirmed that the proposed model can achieve a better performance than that of the traditional WPCNs.

## Appendix A

If $R_{\mathrm{sum}}$ is a concave function, its Hessian is negative semidefinite. To prove Lemma 1, we first derive the Hessian matrix, which is denoted by H and given as follows.

$$H=\left[\begin{array}{ccccccc} \frac{\partial^{2}R_{\mathrm{sum}}}{\partial \alpha_{1}^{2}} & \cdots & \frac{\partial^{2}R_{\mathrm{sum}}}{\partial \alpha_{1}\partial \alpha_{i}} & \cdots & \frac{\partial^{2}R_{\mathrm{sum}}}{\partial \alpha_{1}\partial \alpha_{K}} & \frac{\partial^{2}R_{\mathrm{sum}}}{\partial \alpha_{1}\partial t_{1}} & \frac{\partial^{2}R_{\mathrm{sum}}}{\partial \alpha_{1}\partial t_{2}}\\ \vdots & \ddots & \vdots & \ddots & \vdots & \vdots & \vdots \\ \frac{\partial^{2}R_{\mathrm{sum}}}{\partial \alpha_{i}\partial \alpha_{1}} & \cdots & \frac{\partial^{2}R_{\mathrm{sum}}}{\partial {\alpha_{i}}^{2}} & \cdots & \frac{\partial^{2}R_{\mathrm{sum}}}{\partial \alpha_{i}\partial \alpha_{K}} & \frac{\partial^{2}R_{\mathrm{sum}}}{\partial \alpha_{i}\partial t_{1}} & \frac{\partial^{2}R_{\mathrm{sum}}}{\partial \alpha_{i}\partial t_{2}}\\ \vdots & \ddots & \vdots & \ddots & \vdots & \vdots & \vdots \\ \frac{\partial^{2}R_{\mathrm{sum}}}{\partial \alpha_{K}\partial \alpha_{1}} & \cdots & \frac{\partial^{2}R_{\mathrm{sum}}}{\partial \alpha_{K}\partial \alpha_{i}} & \cdots & \frac{\partial^{2}R_{\mathrm{sum}}}{\partial \alpha_{K}^{2}} & \frac{\partial^{2}R_{\mathrm{sum}}}{\partial \alpha_{K}\partial t_{1}} & \frac{\partial^{2}R_{\mathrm{sum}}}{\partial \alpha_{K}\partial t_{2}}\\ \frac{\partial^{2}R_{\mathrm{sum}}}{\partial t_{1}\partial \alpha_{1}} & \cdots & \frac{\partial^{2}R_{\mathrm{sum}}}{\partial t_{1}\partial \alpha_{i}} & \cdots & \frac{\partial^{2}R_{\mathrm{sum}}}{\partial t_{1}\partial \alpha_{K}} & \frac{\partial^{2}R_{\mathrm{sum}}}{\partial {t_{1}}^{2}} & \frac{\partial^{2}R_{\mathrm{sum}}}{\partial t_{1}\partial t_{2}}\\ \frac{\partial^{2}R_{\mathrm{sum}}}{\partial t_{2}\partial \alpha_{1}} & \cdots & \frac{\partial^{2}R_{\mathrm{sum}}}{\partial t_{2}\partial \alpha_{i}} & \cdots & \frac{\partial^{2}R_{\mathrm{sum}}}{\partial t_{2}\partial \alpha_{K}} & \frac{\partial^{2}R_{\mathrm{sum}}}{\partial t_{2}\partial t_{1}} & \frac{\partial^{2}R_{\mathrm{sum}}}{\partial {t_{2}}^{2}} \end{array}\right]$$

where the elements are given as follows

$$\begin{array}{cc} \frac{\partial^{2}R_{\mathrm{sum}}}{\partial \alpha_{m}^{2}} & =-W\frac{\gamma_{m}^{2}t_{2}}{\ln2{\left[t_{2}+\sum_{i=1}^{K}\left(t_{1}-\alpha_{i}\right)\gamma_{i}\right]}^{2}},\\ \frac{\partial^{2}R_{\mathrm{sum}}}{\partial \alpha_{m}\partial \alpha_{n}} & =-W\frac{\gamma_{m}\gamma_{n}t_{2}}{\ln2{\left[t_{2}+\sum_{i=1}^{K}\left(t_{1}-\alpha_{i}\right)\gamma_{i}\right]}^{2}},m\neq n,\\ \frac{\partial^{2}R_{\mathrm{sum}}}{\partial \alpha_{m}\partial t_{1}} & =W\frac{\gamma_{m}t_{2}\left(\sum_{i=1}^{K}\gamma_{i}\right)}{\ln2{\left[t_{2}+\sum_{i=1}^{K}\left(t_{1}-\alpha_{i}\right)\gamma_{i}\right]}^{2}},\\ \frac{\partial^{2}R_{\mathrm{sum}}}{\partial \alpha_{m}\partial t_{2}} & =-W\frac{\gamma_{m}\left(\sum_{i=1}^{K}\left(t_{1}-\alpha_{i}\right)\gamma_{i}\right)}{\ln2{\left[t_{2}+\sum_{i=1}^{K}\left(t_{1}-\alpha_{i}\right)\gamma_{i}\right]}^{2}},\\ \frac{\partial^{2}R_{\mathrm{sum}}}{\partial {t_{1}}^{2}} & =-W\frac{t_{2}{\left(\sum_{i=1}^{K}\gamma_{i}\right)}^{2}}{\ln2{\left[t_{2}+\sum_{i=1}^{K}\left(t_{1}-\alpha_{i}\right)\gamma_{i}\right]}^{2}},\\ \frac{\partial^{2}R_{\mathrm{sum}}}{\partial t_{2}^{2}} & =-W\frac{{\left[\sum_{i=1}^{K}\left(t_{1}-\alpha_{i}\right)\gamma_{i}\right]}^{2}}{\ln2{\left[t_{2}+\sum_{i=1}^{K}\left(t_{1}-\alpha_{i}\right)\gamma_{i}\right]}^{2}t_{2}},\\ \frac{\partial^{2}R_{\mathrm{sum}}}{\partial t_{1}\partial t_{2}} & =W\frac{\left(\sum_{i=1}^{K}\gamma_{i}\right)\left[\sum_{i=1}^{K}\left(t_{1}-\alpha_{i}\right)\gamma_{i}\right]}{\ln2{\left[t_{2}+\sum_{i=1}^{K}\left(t_{1}-\alpha_{i}\right)\gamma_{i}\right]}^{2}}. \end{array}$$

For any given real vector $x={\left[x_{1},\cdots ,x_{K},x_{K+1},x_{K+2}\right]}^{T}$ , we derive that

$$\begin{array}{c} x^{T}Hx=-\frac{W{\left[t_{2}\left(\left(\sum_{i=1}^{K}\gamma_{i}x_{i}\right)-\left(\sum_{i=1}^{K}\gamma_{i}\right)x_{K+1}\right)-\left(\sum_{i=1}^{K}\gamma_{i}\left(t_{1}-\alpha_{i}\right)\right)x_{K+2}\right]}^{2}}{\ln2{\left[t_{2}+\sum_{i=1}^{K}\left(t_{1}-\alpha_{i}\right)\gamma_{i}\right]}^{2}t_{2}}\leq 0, \end{array}$$

where $t_{2}\geq 0$ . This thus proves Lemma 1.
